# Concomitant epidural and longitudinal anterior spinal artery contrast spread in a lumbar transforaminal epidural steroid injection (TFESI)

**DOI:** 10.1016/j.inpm.2024.100523

**Published:** 2024-12-02

**Authors:** Philip J. Koehler, Paul M. Kitei, David S. Stolzenberg, Elaine H. Hatch

**Affiliations:** a Department of Rehabilitation, Thomas Jefferson University Hospital, Philadelphia, PA, USA; b Department of Physical Medicine and Rehabilitation, Rothman Orthopaedic Institute, Philadelphia, PA, USA

**Keywords:** Pain, Epidural steroid injection, Fluoroscopy, Lumbar radiculopathy, Radiculomedullary artery

## Abstract

A 78-year-old female with a remote history of L3-4 decompression and fusion presented with several months of low back and radicular leg pain. MRI revealed moderate L2-L3 spinal canal stenosis, ligamentum flavum infolding, moderate bilateral foraminal stenosis, and a grade I retrolisthesis. A right sided L2-L3 TFESI was performed using multiplanar fluoroscopic imaging with a subpedicular supraneural approach. During live iodinated contrast injection, imaging revealed concomitant epidural and central arterial contrast spread. The needle was retracted and repeat live fluoroscopic imaging demonstrated no vascular uptake. Desired epidural and nerve root contrast spread remained in place with repeat still imaging. Dexamethasone and lidocaine were then injected. The patient suffered no adverse events. This case demonstrates that during a lumbar TFESI, it is possible to have an inadvertent arterial injection with desired epidural contrast spread, despite appropriate needle placement. It emphasizes the importance of necessary precautions, including real-time live fluoroscopy, in order to detect arterial uptake before the delivery of injectate. Without live fluoroscopy, optimal epidural flow at the targeted level can distract interventionalists from the fleeting vascular flow multiple vertebral levels away and risks continuation of the procedure with delivery of injectate.

**What is Known**. There are established safety precautions to decrease the risk of arterial complication during transforaminal epidural steroid injections such as use of non-particulate steroids, live fluoroscopy, needle approach, digital subtraction imaging, extension tubing, and spinal level of injection.

**What is New**. This case demonstrates a unique contrast pattern during a lumbar TFESI which reinforces the necessary safeguards to prevent neurologic complications. During initial live contrast injection, the observation for vascular flow should be prioritized above optimal spread of into the epidural space, choosing safety over efficiency.

## 1 Introduction

Transforaminal epidural steroid injections (TFESI) are commonly performed to treat radicular pain and are generally regarded as safe procedures. However, rare and devastating complications can occur [1]. Inadvertent vascular injection during TFESI is suggested to increase risk of ischemic mediated spinal cord injury (SCI) [[2], [3], [4]]. Traditionally, the supraneural, subpedicular approach targeting the area between the pedicle and nerve root, known as the “safe triangle”, has been regarded as the optimal approach in order to avoid nerve injury. Despite this, the subpedicular area has been shown to be highly vascularized and contains the highest percentage of arteries. Thus, this approach spares spinal nerves from injury but increases proximity to vasculature. The anterior-superior quadrant contains the majority of the thoracolumbar radicular branches which supply anterior radiculomedullary arteries (ARMA). The ARMAs communicate with longitudinal anterior spinal arteries that supply the spinal cord [2,4,5]. The most well-known ARMA is the Artery of Adamkiewicz, which is the largest intradural blood supply to the anterior spinal cord. The ARMAs enter the spinal canal adjacent to the exiting spinal nerve, then in a classic hairpin like loop, wind along the nerve root before joining the anterior spinal artery [[6], [7], [8]]. The vertebral levels of ARMAs vary, some studies show 83.9 % arise from T12-L3 and 85 % from T9-L2, yet paraplegia has resulted from injections performed at lower lumbar levels [2,9,10]. The mechanism of ischemic spinal cord injury following TFESI is unknown but it is hypothesized to be caused by inadvertent arterial embolization, thrombosis, or needle-induced arteriospasm [2,4,8]. Intravascular injection can potential create retrograde or anterograde flow of injected medication leading to compromise or occlusion of the anterior spinal artery. Particular care should be taken when attempting left sided injections between the levels of T8-L1 as it has been estimated that approximately 90 % of the Artery of Adamkieicz occur in this location [8]. Given the variability of vasculature and potential for catastrophic complications following intravascular injection, necessary precautions should be utilized to avoid or at least recognize intravascular injection which has been estimated to occur between 8.1 % and 12 % of all fluoroscopically guided lumbar TFESI with intravenous being more common than intraarterial [11,12]. In this case report, we present a lumbar TFESI with aberrant contrast flow demonstrating concomitant epidural and longitudinal anterior spinal artery contrast spread observed during real-time live fluoroscopy. We discuss the importance of injecting contrast medium under real-time live fluoroscopy with an anteriorposterior (AP) view before the delivery of any medication that could have detrimental effects if injected intravascularly as well as additional necessary precautions.

## 2 Case presentation

**Fig. 1:**
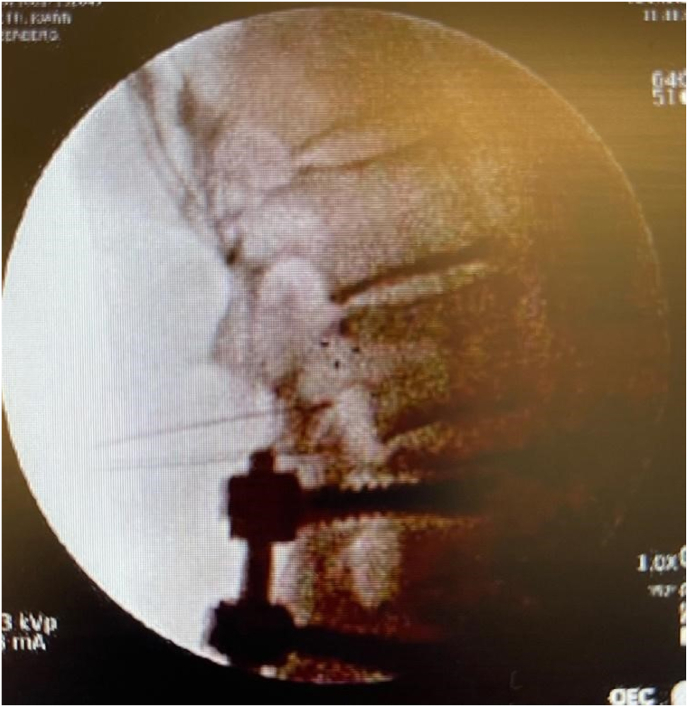
Needle placement with location in the superior and posterior aspect of the L2-L3 foramen.

**Fig. 2:**
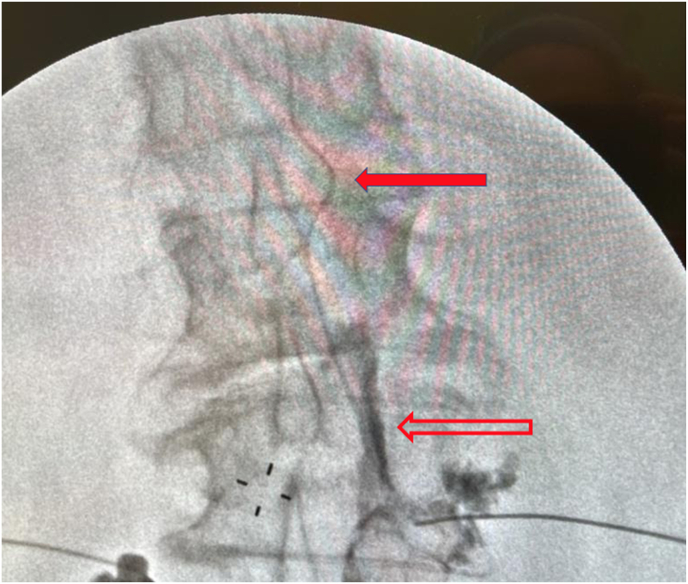
Still shot of live contrast spread during a right-sided L2-L3 TFESI above the level of the L3-L4 fusion. The solid arrow displays central arterial contrast spread and the hollow arrow indicates epidural contrast spread.

A 78-year-old female with a remote history of L3-4 decompression and fusion was referred to our multispecialty spinal institute for low back and radicular leg pain. At the time of evaluation, she had a multiple month history of back pain with claudicatory radiation bilaterally from the gluteal region through lateral thigh to the knee that had not responded to conservative care. Her MRI (Magnetic Resonance Imaging) of the lumbar spine demonstrated moderate L2-L3 spinal canal stenosis secondary to a disc bulge, ligamentum flavum infolding, and a grade I retrolisthesis. An L2-L3 subpedicular supraneural right sided TFESI was performed using a 22 gauge 5 inch length short bevel Quincke needle. Needle placement was performed using multiplanar fluoroscopic imaging with precise guidance to the superior and posterior aspect of the L2-L3 foramen (Fig. 1) and 6 o’clock position under the L2 pedicle. A still image from the live iodinated contrast injection is seen in Fig. 2. The solid arrow displays central arterial contrast spread and the hollow arrow indicates epidural contrast spread. The needle was retracted and repeated live fluoroscopic imaging demonstrated no vascular uptake, with only epidural and nerve root flow that remained in place with repeat still imaging. Dexamethasone and lidocaine were then injected. The patient suffered no adverse events. The patient had transient relief with the injection and later elected to undergo surgical management.

## 3 Discussion

This case demonstrates that during a lumbar TFESI it is possible to have an inadvertent arterial injection while at the same time having the desired epidural contrast spread. Despite the needle being appropriately placed using a subpedicular supraneural approach it is possible to have aberrant contrast flow. The artery we presumed to be visualized was a central longitudinal anterior spinal artery it was straight, trended caudally and toward the midline over three vertebral segments. If this was not an anterior spinal artery it is likely a large reinforcing vessel to the anterior spinal artery.

The case emphasizes the importance of necessary precautions to detect arterial uptake, particularly contrast medium injection under not only still shot but real-time live fluoroscopy with an anteriorposterior (AP) view before the delivery of medication. Still shot frame exposure has advantages of allowing for analysis of injectate flow while minimize radiation exposure. However, in this case, if real-time live fluoroscopy were not used, the artery may not have been visualized as arterial flow is typically brisk. Moreover, a still shot depicting the optimal epidural flow without the arterial flow may have created a false sense of safety and may have encouraged continuation of the procedure with delivery of injectate.

During initial live contrast injection, the observation for vascular flow should be prioritized above optimal spread of contrast into the epidural space, as the epidural contrast will remain, whereas the vascular flow will clear upon stopping the contrast injection. This prioritizes safety over speed. The contrast injection should be performed while optimizing the amount of X-ray collimation in order to both minimize radiation exposure while still including a large enough view to see multiple segments above and below the target site. It should also be performed slowly to allow enough time for visualization of vascular flow, delineate between venous and arterial uptake, in addition to intrathecal and subdural flow. Arterial flow is typically of small caliber, brisk, dissipates rapidly, can spread multiple vertebral levels quickly and be easily missed if not watching closely. Venous flow is characterized by narrow bands with smooth tube-like margins, having uniform density within each band, “serpiginous patterns” communicating with one another via tributaries or branches. It also typically flows medial to lateral as opposed to arterial flow which is the opposite. In this case, the optimal epidural flow at the targeted level may have distracted the physician from the arterial flow multiple vertebral levels away.

The risk presented with inadvertent artery injection can be further minimized with non-particulate steroids. In comparison to non-particulate steroids, when injected intravascularly, particulate steroids have a larger size and tendency to aggregate which inordinately increases the risk of embolization, arterial occlusion, and ischemia [13]. The clinical risk of particulate steroids was corroborated in a comparison study with pigs with direct vertebral artery injection of particulate and non-particulate steroids. There was respiratory arrest in the entirety of the group that received particulate steroids while the group that received non-particulate steroids suffered no neurologic sequelae. MRI and histopathologic analysis confirmed cerebrovascular insult most commonly in the brainstem of the deceased animals [3]. These findings as well as an additional safety study in rats combined with efficacy studies demonstrating non-inferiority to particulate steroids, resulted in the use of non-particulate steroids becoming the recommendation across multiple societal guidelines for TFESIs [14,15].

Despite the proper selection of non-particulate steroids during TFESI, the risk of complication with intravascular injection of local anesthetic is not trivial. Injection into neurovascular structures can result in transient neurologic deficits for the duration of local anesthetic effect. The transient effects of local anesthesia on the spinal cord are unable to be immediately differentiated from cord ischemia and necessitate emergent workup. This is the premise of utilizing a “test-dose” of local anesthetic to further rule out arterial uptake, where normal motor function assessment on the contralateral side after 2 minutes is confirmed prior to injecting corticosteroid, especially if using particulate steroid.

Other procedural considerations are the use of digital subtraction imaging (DSI) when available. In opposition to cervical TFESI, DSI is currently not considered essential for safety on lumbar TFESI and does increase radiation exposure substantially. However, it has been shown to increase the detection of vascular uptake [16]. One prospective study showed 60 % sensitivity to vessel detection when DSI was used as compared to 20 % sensitivity with aspiration alone [17]. Furthermore, a meta-analysis showed a 30 % improvement in vascular detection with DSI in comparison to real time fluoroscopy [18]. Therefore, judicious use of contrast and DSI could be considered for utilization in lumbar TFESI where arterial injection is more likely, such as the upper lumbar vertebrae. The injection was performed at the L2-L3 level, and it has been well documented that the upper lumbar levels contain higher percentages of ARMAs and by denotation are closer to the anterior spinal arteries theoretically posing a higher risk of embolization [2]. Another consideration is use of DSI when the field is obscured by prior contrast or spinal fusion hardware.

Approach should be considered when attempting to minimize the risk of arterial complications during TFESI. For example, an infraneural approach could be considered which is completed by targeting Kambin’s triangle, defined as a right triangle formed by endplate of the lumbar vertebrae inferiorly, traversing nerve root and dural sac medially with the hypotenuse as the exiting nerve root. This approach has been suggested to be equal in short-term efficacy as compared to a subpedicular approach [19,20]. It offers an alternative method when needle tip positioning in the epidural space is difficult and decreases proximity to ARMA. Nevertheless, in typical anatomy this approach could possibly increase the risk of nerve root injury and intervertebral disc puncture, therefore all standard precautions including extensive pre-procedural review of advanced imaging should be used as anatomic variability makes it difficult to predict artery location using fluoroscopy alone.

Use of extension tubing may further reduce the chance of needle manipulation when exchanging syringes from contrast injectate for steroid injectate. The theoretical risk of vascular injury or injection increases with any needle movement or displacement. This also allows the physicians hands to be out of the field during live contrast injection.

Lastly, although there is a paucity of evidence-based literature on how to proceed after encountering arterial uptake on a TFESI, these authors do not suggest redirecting the needle in the same foramen and potentially encountering the same artery again. Considering these are typically elective procedures, reasonable options are to change the approach to interlaminar or inject a different foramen if the target pathology can still be reached or retract the needle and re-inject live contrast.

## 4 Conclusion

Overall, this image demonstrates a unique contrast pattern during a lumbar TFESI which reinforces the necessary safeguards to prevent neurologic complications. We encourage continued research characterizing arterial location, reporting of adverse outcomes, and studies on risk and efficacy of TFESI. With future research we could imagine different techniques and tools such as DSI becoming important to improving safety.

## Author disclosures

The authors have no competing interests or other financial interests connected with this work. No part of the manuscript or the results have been published elsewhere, or have been submitted for publication elsewhere. All authors are responsible for reported research and have participated in the concept and design, analysis and interpretation of data, drafting or revising, and have approved this manuscript as submitted.

## Data availability statement

The data that support the findings of this study are available from the corresponding author upon reasonable request.

## Declaration of competing interest

The authors declare that they have no known competing financial interests or personal relationships that could have appeared to influence the work reported in this paper.
